# Tristetraprolin Gene Single-Nucleotide Polymorphisms and mRNA Level in Patients With Rheumatoid Arthritis

**DOI:** 10.3389/fphar.2021.728015

**Published:** 2021-09-01

**Authors:** Xiaoke Yang, Bo Chen, Mingyue Zhang, Shengqian Xu, Zongwen Shuai

**Affiliations:** ^1^ Department of Rheumatology and Immunology, The First Affiliated Hospital of Anhui Medical University, Hefei, China; ^2^ Department of Nuclear Medicine, Chaohu Hospital Affiliated to Anhui Medical University, Hefei, China; ^3^ Department of Medical Record Room, Fuyang Hospital of Anhui Medical University, Fuyang, China

**Keywords:** tristetraprolin, TTP, rheumatoid arthritis, polymorphism, expression

## Abstract

To observe and evaluate the correlation between single-nucleotide polymorphisms (SNPs) and messenger RNA (mRNA) level related to tristetraprolin (TTP) in Chinese rheumatoid arthritis (RA). TapMan SNP was used for genotyping analysis in 580 RA patients and 554 healthy people. Association between *TTP* gene polymorphisms (rs251864 and rs3746083) and RA was obtained. Quantitative real-time reverse transcription polymerase chain reaction (qRT-PCR) technology was applied for the detection of TTP mRNA level in peripheral blood mononuclear cells (PBMCs) in 36 RA patients and 37 healthy people. We observed that the allele T of *TTP* rs3746083 increased RA susceptibility (*p* = 0.019). A significant difference was found under the dominant model of rs3746083 (*p* = 0.037). Further analysis showed the allele distribution of rs3746083 was nominally correlated with RF phenotype of RA patients (*p* = 0.045). Nevertheless, the association between *TTP* rs251864 and the incidence of RA was no statistically significant (*p* > 0.05). The TTP expression level in PBMCs of RA patients was significantly reduced (*p* < 0.001). In conclusion, the results of this experiment support that TTP may be involved in the pathogenesis of RA.

## Introduction

Rheumatoid arthritis (RA) is a systemic autoimmune disease characterized by autoantibody production, persistent inflammatory synovitis and progressive destruction of articular cartilage and bone (Jiang et al., 2014). The pathogenesis of RA remains obscure. Although both genetic and environmental factors can cause RA, the genetic components were broadly confirmed to account the principal risk for RA susceptibility (Okada et al., 2014; Okada et al., 2019).

The decay of messenger RNA (mRNA) is associated with many inflammation-related gene expression regulation mechanisms. Conserved mRNA sequence elements found in most inflammation-associated genes are adenylate and uridylate-rich elements (AU-rich elements, AREs) which situated in the 3′untranslated region (3′UTR) of mRNA (Sanduja et al., 2011). Whether RNA-binding protein binds to the ARE will affect the stability of mRNA (Khabar, 2010).

**FIGURE 1:**
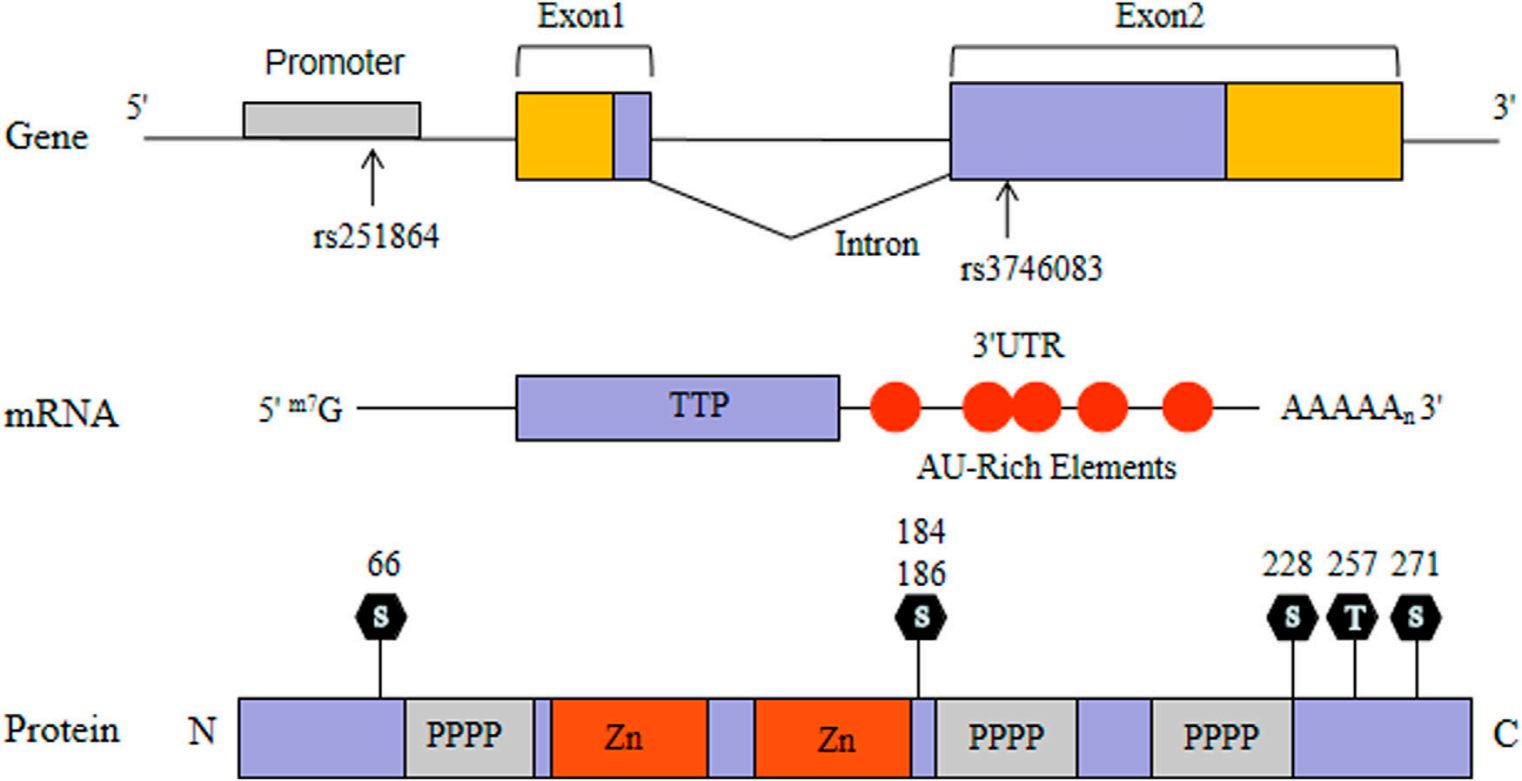
Schematic showing the organization of TTP gene, mRNA, and protein.

Tristetraprolin (TTP) as a RNA-binding protein has been well characterized. TTP, also named Tis11, G0S24 and Nup475, was expressed widely in mammalian cells (Taylor et al., 1991) (Figure 1). TTP-deficient mice exhibited systemic autoimmune inflammatory symptoms consisting of erosive arthritis, cachexia, conjunctivitis, dermatitis and glomerular mesangial thickening (Taylor et al., 1996). The symptoms can be avoided in TTP-deficient mice by injecting with antibodies to tumor necrosis factor-α (TNF-α), suggesting that the inflammatory symptoms are caused by TNF-α elevation (Taylor et al., 1996). In an attempt to clarify this mechanism, Carballo and collegues found that TTP directly binds to the ARE of TNF-α mRNA and degrade it rapidly (Carballo et al., 1998). Later, TTP was identified to bind the AREs and regulate other pro-inflammatory cytokines (Lee et al., 2012; Härdle et al., 2015). TTP expression which partly interferes with the p65 subunit of nuclear factor κB (NF-κB) attenuates NF-κB activity (Schichl et al., 2009). Moreover, TTP could physically interact with histone deacetylases (HDACs) and act as a common suppressor of NF-κB-dependent transcription (Liang et al., 2009). Therefore, TTP not only contribute to regulate the expression of pro-inflammatory gene through its mRNA-decay function, but also participate in the regulation of inflammatory signal transduction. Taking together these findings, it suggests that TTP may be involved in the pathogenesis of RA.

*TTP* gene, also known as *zinc finger protein 36 (ZFP36)*, consists of two exons and one intron, which is located on human chromosome 19q13.1 and about 3,100 bp in length (Guo et al., 2017). After searchimg the relevant literature, we did not find any previous associations between *TTP* gene and RA in the Chinese population. Therefore, we established a two-phase study of TTP in RA, aiming to understand whether the polymorphisms of *TTP* gene (rs251864 and rs3746083) are related to RA susceptibility. Concurrently, the TTP mRNA level in peripheral blood mononuclear cells (PBMCs) was evaluated.

## Materials and Methods

### Patients and Controls

580 RA patients (483 females and 97 males, mean age 51.23 ± 12.71 years) and 554 healthy controls (479 females and 75 males, mean age 50.30 ± 17.80 years) were enrolled to explore whether *TTP* gene polymorphisms are related to disease susceptibility. The diagnosis of RA was met the European League Against Rheumatism (EULAR) 2009 and the American College of Rheumatology (ACR) 1987 revised classification criteria for RA (Arnett et al., 1988). All patients were recruited from the First affiliated Hospital of Anhui Medical University. All healthy individuals had no history of RA or other autoimmune diseases.

And then, 36 RA patients (33 females and three males, mean age 53.67 ± 10.63 years) and 37 healthy controls (35 females and two males, mean age 50.14 ± 9.81 years) were randomly selected from the genotyping samples to investigate the expression of TTP.

The demographic and laboratory parameters were obtained from questionnaires and medical records. According to the 1964 Helsinki Declaration, each subject obtained informed consent. The research scheme was ratified by the Ethics Committee of Anhui Medical University.

### Single-Nucleotide Polymorphisms Genotyping

The genomic DNA samples in peripheral blood cells were lifted in line with the specifications of the Flexi Gene DNA Kit (Qiagen, German) and then we used TapMan SNP to determine genotyping on the EP1 system (Fluidigm, CA, United States). Two SNPs in the *TTP* gene were selected: rs3746083 (39898667C > T) in the second exon and rs251864 (39897293A> G) in the promoter exon. The probes (assay ID) used in this study were as follows: rs3746083 (C_2997179_10) and rs251864 (C_610276_10).

### Quantitative Real-Time Reverse Transcription Polymerase Chain Reaction

Peripheral blood was separated by Ficoll-Hypaque density gradient centrifugation to obtain PBMC. Trizol reagent was used for the extraction of total RNA, and PrimeScript™ RT kit (Takara, Japan) was applied to reverse transcribe the obtained RNA into cDNA.

The quantitative Real-Time Reverse Transcription Polymerase Chain Reaction (qRT-PCR) was performed by ViiA™ 7 Real-Time PCR System (Thermo Fisher, CA, United States). The qRT-PCR primer sequences of TTP gene are sense primer: 5′-GAC​TGA​GCT​ATG​TCG​GAC​CTT-3′ and antisense primer: 5′-GAG​TTC​CGT​CTT​GTA​TTT​GGG​G-3′. Housekeeping gene β-actin (sense primer: 5′-CAC​GAA​ACT​ACC​TTC​AAC​TCC-3′, antisense primer: 5′-CAT​ACT​CCT​GCT​TGC​TGA​TC-3′) was used as internal control. Based on the instructions of SYBR Green (Takara Bio Inc., Japan), we add 0.4 µl sense primer and 0.4 µl antisense primer (10 µM) into the qRT-PCR reaction system to establish the following circulation system: 95°C for 1 min and then 42 cycle under the programs of 95°C for 10 s, 60°C for 30 s and 72°C for 1 min. 2^−△△Ct^ was used to compute the relative expression level of TTP.

### Statistical Analysis

The obtained data are measured by the mean ± standard deviation of their normal distribution, and the non-normal distribution data is represented by the median value and interquartile range. The non-parametric test was applied to analyze the differences in TTP mRNA expression within the groups, and the Spearman rank correlation was applied to test the correlation between the groups.

Using i-square (χ^2^) test or fisher test to test the genotype and allele frequency distribution between groups, and perform odds ratio (OR) and 95% confidence interval (CI) for regression analysis of results, and use dominant model and invisible model for statistical analysis. In addition, the Hardy-Weinberg equilibrium (HWE) test was performed in healthy subjects.

Use SPSS 19.0 software (IBM Corp) to perform statistical analysis on the obtained data. A two-tailed *p* value ≤ 0.05 was regarded as statistically significant.

## Results

### Association of Tristetraprolin Polymorphisms With Rheumatoid Arthritis Susceptibility

The characteristics of RA patients are presented in Table 1. The observed genotype frequencies of rs251864 and rs3746083 were distributed in accordance with HWE in the controls (χ^2^ = 2.039, *p* = 0.153;χ^2^ = 2.435, *p* = 0.119).

**TABLE 1:** The main demographic and clinical characteristics of rheumatoid arthritis patients.

Characteristics	RA patients (N = 580)
Age (years) mean ± SD	51.23 ± 12.71
Sex, female/male	483/97
Age of onset, years, M ± SD	44.34 ± 13.91
Disease duration, years, M ± SD	6.79 ± 7.37
Treatment duration (years) mean ± SD	5.74 ± 7.24
RF-positive no (%)	459(79.1)
Anti-CCP positive no (%)	429(74.0)

RA, rheumatoid arthritis; RF, rheumatoid factor; Anti-CCP, anti-cyclic citrullinated peptide.

Compared with healthy controls, RA patients have a significantly higher frequency of *TTP* rs3746083 T allele (OR = 1.611, 95%CI = 1.078–2.407, *p* = 0.019). In addition, there was a significant difference under the dominant model of rs3746083 (TT + TC versus CC: OR = 1.567, 95%CI = 1.025–2.395, *p* = 0.037). Nevertheless, no association between *TTP* rs3746083 and RA was observed in genotype distribution and recessive model (all *p* > 0.05). Besides, it can be proved that there is no obvious correlation between *TTP* rs251864 polymorphism and RA susceptibility (all *p* > 0.05) (Table 2).

**TABLE 2:** Allele and genotype frequencies of the TTP gene SNPs in RA patients and healthy controls.

SNP	Genotype	RA (n = 580) n(%)	Controls(n = 554) n(%)	OR (95%CI)	*p* Value
rs251864	GG	40(6.9)	45(8.1)	0.850(0.540–1.338)	0.483
	GA	219(37.8)	202(36.5)	1.037(0.810–1.327)	0.774
	AA	321(55.3)	307(55.4)	Reference	
	G	299(25.8)	292(26.4)	0.970(0.805–1.171)	0.745
	A	861(74.2)	816(73.6)	Reference	
Dominant model	GG + GA	259(44.7)	247(44.6)	1.003(0.793–1.268)	0.981
	AA	321(55.3)	307(55.4)	Reference	
Recessive model	GG	40(6.9)	45(8.1)	0.838(0.538–1.305)	0.433
	GA + AA	540(93.1)	509(91.9)	Reference	
rs3746083	TT	6(1.0)	2(0.4)	2.977(0.598–14.818)	0.162
	TC	54(9.3)	36(6.5)	1.488(0.960–2.309)	0.074
	CC	520(89.7)	516(93.1)	Reference	
	T	66(5.7)	40(3.6)	1.611(1.078–2.407)	0.019^a^
	C	1,094(94.3)	1,068(96.4)	Reference	
Dominant model	TT + TC	60(10.3)	38(6.9)	1.567(1.025–2.395)	0.037^a^
	CC	520(89.7)	516(93.1)	Reference	
Recessive model	TT	6(1.0)	2(0.4)	2.885(0.580–14.355)	0.176
	TC + CC	574(99.0)	552(99.6)	Reference	

RA, rheumatoid arthritis; OR, odds ratio; CI, confidence interval.

a Statistically significant (*p* < 0.05).

### Tristetraprolin Polymorphisms and Laboratory Parameters of Rheumatoid Arthritis

We evaluated the associations of rs251864 and rs3746083 SNPs with rheumatoid factor (RF) and anti-cyclic citrullinated peptide (anti-CCP) in RA patients (Table 3). We observed the allele distribution of rs3746083 was nominally correlated with RF phenotype in patients with RA (χ^2^ = 4.029, *p* = 0.045). However, neither the RF nor the anti-CCP were influenced by rs251864 polymorphism (*p* > 0.05).

**TABLE 3:** Association of TTP gene polymorphisms with auto-antibody profiles in patients with RA.

Variable	Genotype frequency, n (%)			χ^2^	*p* Value	Allele frequency, n (%)		χ^2^	*p* Value
rs251864	GG	GA	AA			G	A		
RF									
Positive	26(6.1)	160(37.3)	243(56.6)	2.761	0.251	212(24.7)	646(75.3)	2.118	0.146
Negative	12(9.9)	47(38.9)	62(51.2)			71(29.3)	171(70.7)		
Anti-CCP									
Positive	29(6.3)	180(39.2)	250(54.5)	3.756	0.153	238(25.9)	680(74.1)	0.006	0.937
Negative	9(11.0)	25(30.5)	48(58.5)			43 (26.2)	121(73.8)		
**rs3746083**	**TT**	**TC**	**CC**			**T**	**C**		
RF									
Positive	4(0.9)	34(7.9)	391(91.2)	3.743	0.154	42(4.9)	816(95.1)	4.029	0.045^a^
Negative	2(1.6)	16(13.3)	103(85.1)			20(8.3)	222(91.7)		
Anti-CCP									
Positive	5(1.1)	42(9.2)	412(89.7)	0.286	0.867	52(5.7)	866(94.3)	0.276	0.599
Negative	1(1.2)	9(11.0)	72(87.8)			11(6.7)	153(93.3)		

RA, rheumatoid arthritis; Anti-CCP, anti-cyclic citrullinated peptide; RF, rheumatoid factor.

a Statistically significant (*p* < 0.05).

### Tristetraprolin Expression Level in Rheumatoid Arthritis

**FIGURE 2:**
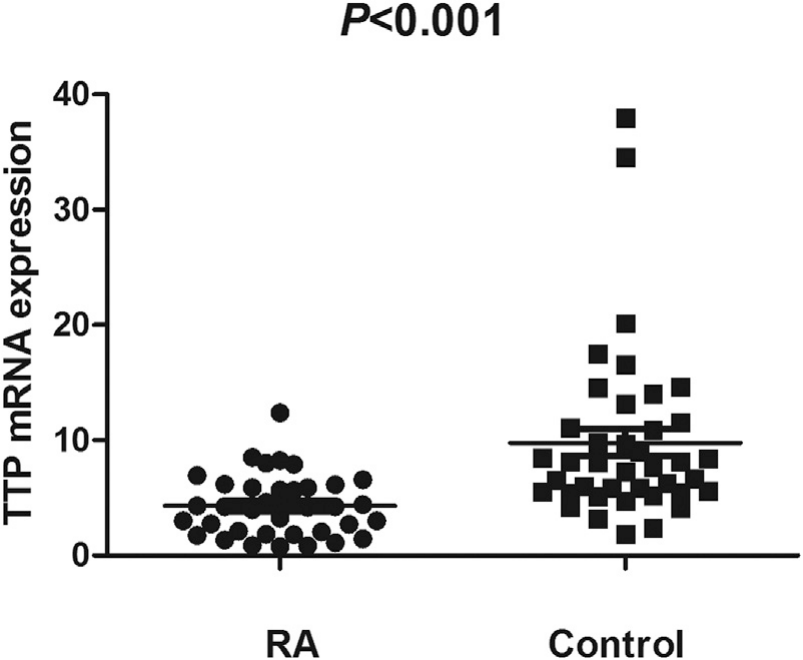
Comparision of TTP mRNA expression level in PBMCs between RA patients and normal controls.

**FIGURE 3:**
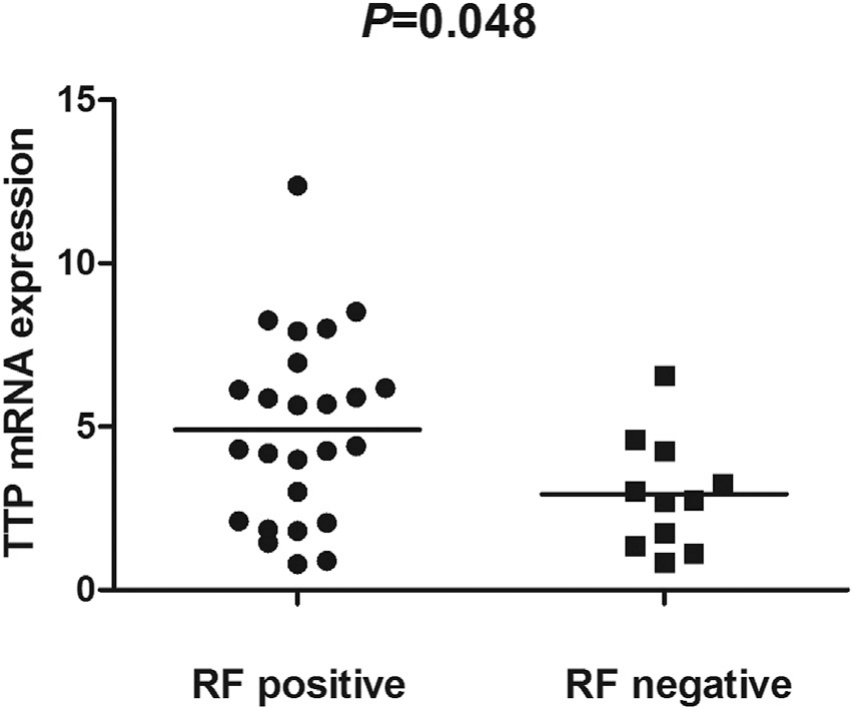
Comparision of TTP mRNA expression level in PBMCs between RF-positive RA patients and RF-negative RA patients.

**FIGURE 4:**
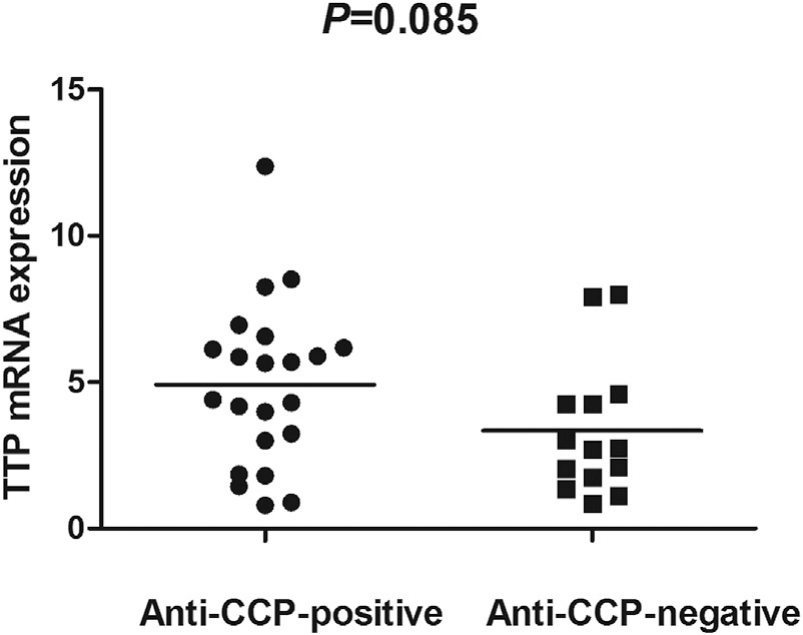
Comparision of TTP mRNA expression level in PBMCs between anti-CCP-positive RA patients and anti-CCP-negative RA patients.

The TTP mRNA expression level in PBMCs of RA patients was nominally significantly lower than healthy people (*Z* = −5.244, *p* < 0.001) (Figure 2). We also analyzed the relationship between TTP mRNA level and RF and anti-CCP of RA. The results showed that TTP mRNA level in RF-negative patients decreased statistically (*Z* = −1.975, *p* = 0.048; Figure 3). However, TTP mRNA expression level did not differ between patients with positive or negative anti-CCP results (*Z* = −1.720, *p* = 0.085; Figure 4). When we divided the RA patients into active and inactive RA, the TTP mRNA level did not show significant difference in active RA compared with inactive RA groups (*p* > 0.05). The correlations of TTP mRNA level with CRP, ESR and DAS28 of RA patients were not statistically significant (all *p* > 0.05).

### Effect of Tristetraprolin Polymorphisms on Tristetraprolin Expression in Rheumatoid Arthritis

We analyzed the associations between the TTP mRNA level of RA patients and the genotypes of rs251864 and rs3746083. However, no statistically significant difference was identified (all *p* > 0.05).

## Discussion

TTP, the most common RNA-binding protein, has been shown to be involved in the regulation of various ARE-containing mRNAs (Lee et al., 2020). The absence of TTP increased the expression and proliferation of its activation markers, and inhibited apoptosis in T cells (Moore et al., 2018). Accumulating evidence pointed to the fact that TTP might be involved in the regulation of autoimmune diseases (Sanduja et al., 2012; Ross et al., 2017; Yoshinaga and Takeuchi, 2019). Considering the role of TTP in immune system and its derivation in autoimmune diseases, analyzing the polymorphism of TTP gene in Chinese RA patients can better understand the role of RA susceptibility. This analysis is very necessary and has practical significance.

SNP is responsible for an individual’s genetic component, including complex traits such as drug response and disease susceptibility (Mullikin et al., 2000). Actually, previous studies have evaluated the role of *TTP* gene polymorphisms in some autoimmune diseases. Carrick et al. (2006) studied the association between *TTP* polymorphisms and RA susceptibility, multiple sclerosis (MS) and psoriasis. Their study demonstrated that the higher incidence of RA in African-Americans was related to the T allele of SNP rs3746083. Subsequently, Suzuki et al. identified a SNP, rs251864, within the TTP promoter region, which is related to the course of RA and treatment response of anti-TNF-α in Japanese RA patients (Suzuki et al., 2008). This study further suggested that rs251864 affected TTP promoter activity, which means that there is a connection between TTP and RA. Here, our current study investigated the association between *TTP* gene rs251864 and rs3746083 polymorphisms and RA in the Chinese population. Our results suggested that *TTP* rs3746083 was a genetic marker significantly related to RA. In addition, we discovered rs3746083 polymorphism was related to RF-positive patients, suggesting that disease prognosis is also associated with the rs3746083 variant.

Simultaneously, we investigated the TTP mRNA expression in PBMCs of RA. Our results indicated that TTP mRNA expression level was lower in RA patients. The decreased TTP mRNA level in RA demonstrated that TTP may be a protective factor for RA, and this result is agreement on the results of a previous study by the Japanese population (Sugihara et al., 2007). Moreover, the presence of positive for RF was associated with TTP mRNA level in RA patients. These results indicated that TTP mRNA level might play a role in the phenotype of RA.

The exact mechanism underlying the effects of TTP is unclear. TTP is expressed in several immune-related tissues and cells, implying a potential role of TTP in the immune system. Early research also demonstrated that TTP was involved in regulating the production of multiple inflammatory cytokines, thereby affecting the immune response, including TNF-α, interleukins (ILs) and interferons (IFNs) (Brooks and Blackshear, 2013). The macrophages from TTP-deficient mice exhibit an apparent primary tendency to excess TNF-α production, which indicates the role of TTP in the biosynthesis and/or release of TNF-α in this cell type (Carballo et al., 1997). IL-12 is one of the ILs families and regulates immune response. Previous study shows that TTP down-regulates IL-12 at transcriptional level through the NF-κB pathway (Gu et al., 2013). IFN-γ plays a vital role in the immune regulation and enhances antiviral ability. TTP mediates the degradation of IFN-γ by binding to the functionally ARE of IFN-γ mRNA. It implied that TTP mediates IFN-γ mRNA decay (Ogilvie et al., 2009). Modification after translation, especially phosphorylation, has a great influence on the anti-inflammatory effect of TTP (Cao et al., 2007). Replacing TTP phosphorylation sites conferred significantly protect mice from inflammatory arthritis. It can be used in treatment of inflammatory diseases by changing the degree of phosphorylation (Ross et al., 2015). This may give us a signal: immune and inflammatory diseases can be treated by stimulating the expression of TTP. In addition, the results of various mouse models are mostly in line with the findings of *in vitro* studies, which is worthy of our attention. Now people have a deeper understanding of the therapeutic effects of TTP through these experiments (Phillips et al., 2004; Patial and Blackshear, 2016). Furthermore, the functional consequences of SNPs rs251864 and rs3746083 have been consistently thought to have a negative impact on the transcription, translation and stability of TTP mRNA. All of these make *TTP* an interesting RA candidate gene.

To our best knowledge, this is the first study to prove that TTP dysregulation might be related to the pathogenesis and development of RA, and the rs3746083 polymorphism of the *TTP* gene may be protective of RA in a Chinese population. However, several limitations should be acknowledged. First of all, the sample size of the case-control study we included is relatively small, thus resulting in moderate statistical power for rs3746083. Secondly, the data in this study is based on the hospital’s case-control, and selection bias was inevitable. Thirdly, the potential impact of environmental factors cannot be ruled out. Finally, we have not performed a functional study of TTP.

## Data Availability

The original contributions presented in the study are included in the article/supplementary material, further inquiries can be directed to the corresponding author.
